# Successful treatment of methemoglobinemia in an elderly couple with severe cyanosis: two case reports

**DOI:** 10.1186/1752-1947-6-290

**Published:** 2012-09-11

**Authors:** Ying-Fu Su, Li-Hua Lu, Tai-Hao Hsu, Shih-Liang Chang, Rong-Tsung Lin

**Affiliations:** 1 Department of Emergency Medicine, Tungs’ Taichung Metro Harbor Hospital, Taichung County, Taiwan, ROC; 2 Department of Medicinal Botanicals and Health Applications, College of Biotechnology and Bioresources, Da-Yeh University, Changhua County, Taiwan, ROC; 3 Department of Occupational Safety and Health, Jen-Teh Junior College of Medicine, Nursing and Management, Houlong, Miaoli County, Taiwan, ROC; 4 Tungs’ Taichung Metro Harbor Hospital, No.699, Sec.1, Chungchi Road, Wuchi, Taichung, 435, Taiwan, ROC

## Abstract

**Introduction:**

Methemoglobinemia should be considered in all cyanotic patients who remain unresponsive to oxygen therapy. Rapid diagnosis is very important in emergency cases. Here, we present the cases of two patients, a married couple, admitted to our hospital with methemoglobinemia after exposure to sodium nitrite.

**Case presentation:**

Two patients, a married couple, presented with methemoglobinemia. The 72-year-old Taiwanese man and 68-year-old Taiwanese woman were referred to our hospital with dizziness and tachypnea. On examination, their mucous membranes were cyanotic, and their blood samples showed the classic ‘chocolate brown’ appearance. The man also reported having experienced twitching of his right arm for a few minutes before arrival at the hospital. The symptoms of both patients failed to improve in response to supplemental oxygen delivered via oxygen masks, although the arterial blood gas data of these patients were normal and their pulse oximetry showed oxyhemoglobin levels of approximately 85%. A carbon monoxide-oximeter showed that the man’s methemoglobin concentration was 48.3%, and the woman’s was 36.4%. Methylene blue (100mg) was administered intravenously to both patients, and their symptoms improved dramatically. They were admitted to the intensive care unit and discharged three days later, without neurological sequelae.

**Conclusion:**

Severe methemoglobinemia is a life-threatening condition and, if untreated, may result in death. Early diagnosis and appropriate antidotal treatment are crucial in treating this emergency situation.

## Introduction

Hemoglobins can be classified into two groups: those capable of binding oxygen (O_2;_ normal hemoglobins) and those incapable of binding O_2_ (dyshemoglobins). Methemoglobin represents the oxidized form (Fe^3+^-Hb) of hemoglobin, which is incapable of carrying oxygen [1]. Acquired methemoglobinemia can result from exposure to a wide range of drugs and chemicals, including nitrites and other oxidants. Signs and symptoms of methemoglobinemia, which may correlate with measured methemoglobin level, include headache, tachypnea, cyanosis, and changes in mental status. Arterial blood gas and pulse oximeter measurements offer limited diagnostic help and may be misleading. We present the case of two patients who, to avoid a lethal outcome, were diagnosed within an hour as having acquired methemoglobinemia. Before the admission, our patients had ingested food containing large amounts of sodium nitrite, as determined using a specific test paper (see below). Further evidence to support the diagnosis was obtained through physical examination and successful treatment with methylene blue. This case provides a successful model for emergency treatment for methemoglobinemia.

## Case presentations

### Case 1

**Figure 1:**
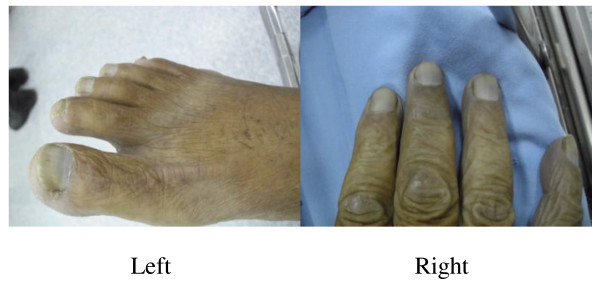
The male patient’s foot (left) and hand (right) showed bluish coloration; cyanotic nail beds can be seen.

A 72-year-old Taiwanese man was referred to our hospital with complaints of tachypnea and dizziness after lunch. Our patient had no notable history of orthopnea, chest pain, palpitations, or any other systemic illness. He had a clinical history of consuming bamboo shoot soup at lunch; this soup had a ‘rusty odor’. On arrival at our emergency department, our patient’s lips were pale grey, his nail beds were cyanotic (Figure 1), and he experienced an involuntary twitch in his right arm. Vital signs at presentation were as follows: pulse rate, 90 beats/min; respiratory rate, 28breaths/min; and blood pressure, 100/60mmHg. Bilateral breath sounds were clear on auscultation of his chest. His blood samples appeared chocolate brown; the color did not change when oxygen was given through a facemask. On 8L/min of supplemental oxygen, pulse oximetry showed a saturation of 87%, and arterial blood gas analysis showed a partial pressure of O_2_ of 95.8mmHg (80 to 105mmHg) and a hemoglobin O_2_ saturation level of 87.8%. Electrocardiography showed diffuse ST depression, although his cardiac enzyme levels were within the normal range.

### Case 2

Our second patient was a 68-year-old Taiwanese woman who attended our hospital with the same complaints as her husband, as described above. Like her husband, she had no relevant past medical history. On inspection, she had mild generalized cyanosis, pale grey lips, and bluish nail beds. Her vital signs at presentation were as follows: pulse rate, 90 beats/min; respiratory rate, 27breaths/min; and blood pressure, 96/66mmHg. Her chest was clear on auscultation. Her blood samples were also of the classic chocolate-brown color indicative of methemoglobinemia. On 8L/min of supplemental oxygen, pulse oximetry showed an oxygen saturation of 88%, and arterial blood gas analysis showed a partial pressure of O_2_ of 172.9mmHg (80 to 105mmHg) and a hemoglobin O_2_ saturation level of 96.3%. As was the case in our male patient, electrocardiography showed diffuse ST depression, although her cardiac enzyme levels were within the normal range.

**Figure 2:**
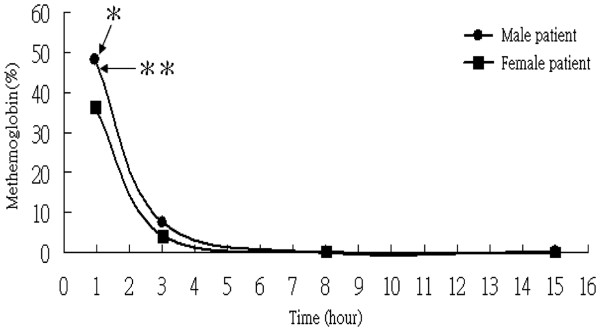
The concentrations of methemoglobin over the first 15 h after sodium nitrite intake. * Represents the time at measurement of methemoglobin level (<1 h) while the patients were in the department of emergency medicine. ** Represents the timing of intravenous methylene blue infusion.

A disparity was found between the symptoms observed clinically and pulse oximeter readings and calculated oxygen saturations. Taking into consideration that pulse oximetry and arterial blood gas results may be misleading when more than two types of hemoglobin are present, direct measurements of oxyhemoglobin were taken using a carbon monoxide (CO)-oximeter. The methemoglobin concentration of our male patient was 48.3% (normal levels, <1% to 2%), and that of his wife was 36.4% (Figure 2). Both patients were initially treated with 100% oxygen through non-rebreather masks, gastric lavage, and administration of activated charcoal. However, their symptoms worsened with an increasingly bluish appearance of the hands, feet, and lips, as well as deterioration of consciousness in the case of the male patient. Antidotal therapy with 100mg methylene blue was administered intravenously over 5 minutes. The clinical conditions of both patients improved dramatically within 30 minutes, along with a gradual resolution of the cyanotic discoloration. Three hours after the methylene blue injection, our patients were no longer cyanotic. The methemoglobin level of the man was 7.5% and that of the woman was 4.2% (Figure 2).

**Figure 3:**
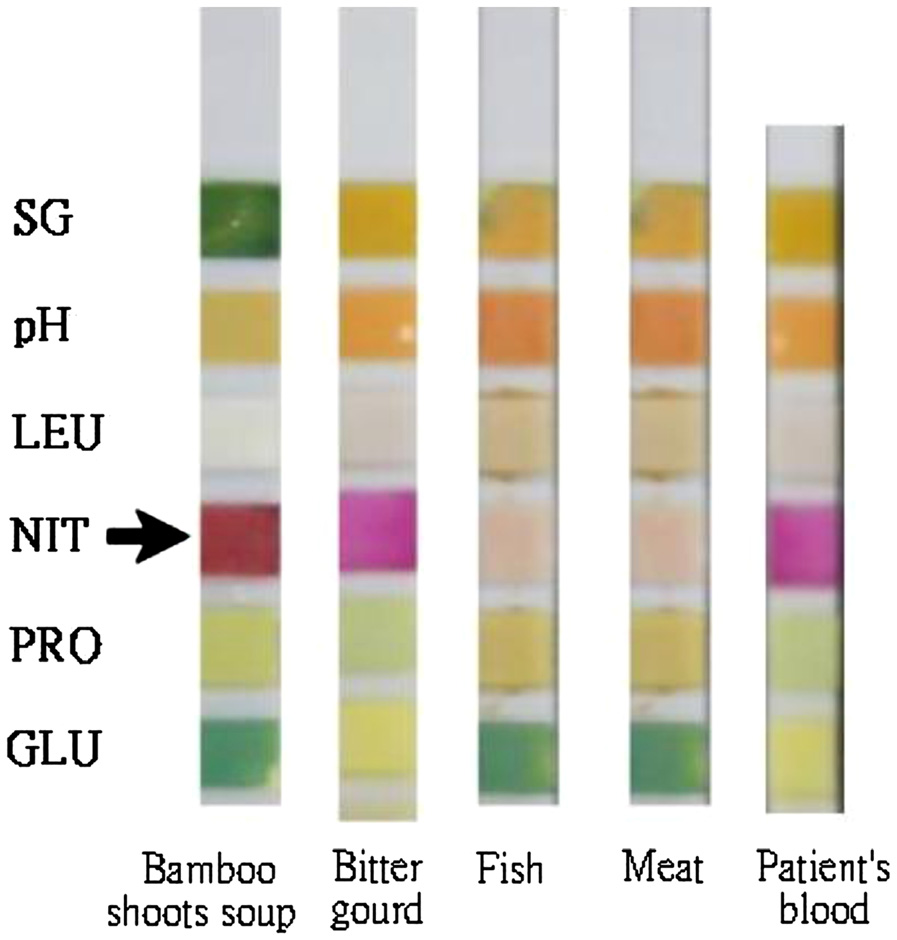
**A strong positive reaction for nitrite was found in the bamboo shoot soup (arrow) when compared to other food by Roche Combur**^10^**Test® M test strips.** A weakly positive reaction of nitrite was found in patient’s blood by using the same kind of test strip. NIT (arrow) = nitrite; SG = specific gravity; pH = hydrogen ion (H+) concentration; LEU = leukocytes; PRO = protein; GLU = glucose.

The bamboo shoot soup, which was thought to be the source of poisoning, was sent to our laboratory for detection of nitrite and any other potential toxicological agents. A strong positive nitrite reaction was found in the soup on testing with Roche Combur^10^ Test® M test strips (Roche Diagnostics GmbH, Mannheim, Germany) based on the principle of the Griess reaction [2]. A weakly positive nitrite reaction was also noted in our patients' blood (Figure 3). The soup was sent to the National Poison Control Center laboratory, and the nitrite level was found to be as high as 6076 μg/mL. After further investigation, the patients’ daughter-in-law admitted that she had intentionally added sodium nitrite, which is used as an industrial bleaching agent, into the soup, causing the life-threatening methemoglobinemia of the couple. 

## Discussion

Methemoglobinemia is a rare cause of cyanosis which should be promptly recognized and well treated. Some cases of acquired methemoglobinemia may result from exposure to certain drugs during medical testing. The symptoms of methemoglobinemia can range in severity from dizziness to coma. Patients may present with cyanosis when the methemoglobin concentration reaches levels of approximately 10% of the total hemoglobin level [3]. In normal circumstances, the enzyme nicotinamide adenine dinucleotide-methemoglobin reductase reduces methemoglobin to hemoglobin, preventing the accumulation of methemoglobin. If this usual mechanism is overwhelmed by exogenous oxidative stress, acquired methemoglobinemia ensues. A second enzymatic pathway uses nicotinamide adenosine dinucleotide phosphate and nicotinamide adenosine dinucleotide phosphate-methemoglobin reductase, which is important for the antidotal effect of methylene blue when administered exogenously [4]. Patients with methemoglobinemia tend to present with a more serious condition than anemic patients, who show a similar reduction in oxygen-carrying capacity due to a leftward shift in the oxyhemoglobin dissociation curve [5]. Profound cyanosis incompatible with the degree of respiratory distress, especially where cyanotic symptoms are unresponsive to oxygen therapy, should raise the suspicion of methemoglobinemia. This diagnosis is supported by the chocolate-brown coloration of blood samples obtained from these patients, and the diagnosis may be confirmed by the results of CO-oximeter.

Nitrites and aniline derivatives have been reported to be among the chemical agents that most commonly cause methemoglobinemia [6]. A review of the literature showed that the reported lethal dose of sodium nitrite in adults is approximately 2.6g [7]. Our two patients showed recovery from the effects of the consumption of almost 3g of sodium nitrite from the soup. The onset of methemoglobinemia occurs usually within 20 to 60 minutes of chemical exposure, although the clinical evolution is difficult to predict concerning these toxic agents. Generally, 10% to 15% methemoglobin saturation produces obvious cyanosis [8]. Methemoglobin concentrations of 50% to 60% impair oxygen delivery, resulting in myocardial ischemia, depressed mental status and seizures, as observed in the case of our patients.

In our patients, the difference between oxygen saturation measured by the pulse oximeter and blood gas analysis led us to consider the possibility of dyshemoglobinemia. The oxygen saturation measured by pulse oximetry typically presents values of around 85% in patients with methemoglobinemia [9]. When patients have significantly elevated methemoglobin levels (>20%), the pulse oximeter falsely indicates high levels of oxygen saturation. Arterial blood gas analysis may also be initially deceptive, because the partial pressure of O_2_, as a measure of dissolved oxygen, is normal. Thus, extrapolation of this figure to predict the expected oxygen saturation will provide a falsely elevated result. The best definitive diagnostic test is multiple wavelength CO-oximetry, an *in vitro* spectrophotometric method that is capable of differentiating between oxy-, deoxy-, met- and carboxyhemoglobin [9].

In the case of patients diagnosed with methemoglobinemia, the optimum treatment is adequate oxygen delivery and appropriate antidotal therapy. Methylene blue is indicated as the first-line antidotal therapy for patients with severe methemoglobinemia. Although successful treatment with plasma exchange therapy, hyperbaric oxygen therapy and ascorbic acid has also been reported, these therapies should be considered as second-line treatments for patients unresponsive to methylene blue. As the methemoglobin level falls, the most severe signs and symptoms will be the first to resolve. As observed in our patients, symptoms of dyspnea and depressed mental status subsided within 30 minutes of methylene blue injection. Cyanosis usually resolves somewhat later, after the levels of methemoglobin have fallen to below 1.5g/dL. The initial dose of methylene blue is 1 to 2mg/kg intravenously. If symptoms of hypoxia fail to subside, the same dose may be repeated within 1 hour [10].

## Conclusions

Methemoglobinemia should be considered in all patients with cyanosis who are unresponsive to oxygen therapy. Rapid diagnosis and early intervention with antidotal therapy should prevent a fatal outcome. Moreover, the simple Roche Combur^10^ Test® M test strips can provide rapid and accurate evidence of the possible presence of nitrites in human body fluids. The treatment of choice is intravenous methylene blue infusion, which should be available in all emergency departments.

## Consent

Written informed consent was obtained from the patients for publication of this manuscript and accompanying images. A copy of the written consent is available for review by the Editor-in-Chief of this journal.

## Competing interests

The authors declare that they have no competing interests.

## Authors’ contributions

YFS treated the patients and wrote the case report. LHL saw the patients when they were admitted to hospital. THH and SLC were responsible for manuscript editing and advice on the literature review. RTL treated the patients, supervised the writing, and reviewed the final version of this manuscript. All authors read and approved the final manuscript.
